# Factors Associated With Adherence to Blood Pressure Measurement Recommendations at Pediatric Primary Care Visits, Minnesota and Colorado, 2007–2010

**DOI:** 10.5888/pcd12.140562

**Published:** 2015-07-23

**Authors:** Emily D. Parker, Alan R. Sinaiko, Matt F. Daley, Elyse O. Kharbanda, Nicole K. Trower, Heather M. Tavel, Nancy E. Sherwood, David J. Magid, Karen L. Margolis, Patrick J. O’Connor

**Affiliations:** Author Affiliations: Elyse O. Kharbanda, Nicole K. Trower, Nancy E. Sherwood, Karen L. Margolis, Patrick J. O’Connor, HealthPartners Institute for Education and Research, Minneapolis, Minnesota; Alan R. Sinaiko, Department of Pediatrics, University of Minnesota, Minneapolis, Minnesota; Matt F. Daley, Heather M. Tavel, David J. Magid, Institute for Health Research, Kaiser Permanente Colorado, Denver, Colorado.

## Abstract

**Introduction:**

Elevated blood pressure in childhood may predict increased cardiovascular risk in young adulthood. The Task Force on the Diagnosis, Evaluation and Treatment of High Blood pressure in Children and Adolescents recommends that blood pressure be measured in children aged 3 years or older at all health care visits. Guidelines from both Bright Futures and the Expert Panel of Integrated Guidelines for Cardiovascular Health and Risk Reduction in Children and Adolescents recommend annual blood pressure screening. Adherence to these guidelines is unknown.

**Methods:**

We conducted a cross-sectional study to assess compliance with blood pressure screening recommendations in 2 integrated health care delivery systems. We analyzed electronic health records of 103,693 subjects aged 3 to 17 years. Probability of blood pressure measurement documented in the electronic health record was modeled as a function of visit type (well-child vs nonwell-child); patient age, sex, race/ethnicity, and body mass index; health care use; insurance type; and type of office practice or clinic department (family practice or pediatrics).

**Results:**

Blood pressure was measured at 95% of well-child visits and 69% of nonwell-child outpatient visits. After adjusting for potential confounders, the percentage of nonwell-child visits with measurements increased linearly with patient age (*P* < .001). Overall, the proportion of children with annual blood pressure measurements was high and increased with age. Family practice clinics were more likely to adhere to blood pressure measurement guidelines compared with pediatric clinics (*P* < .001).

**Conclusion:**

These results show good compliance with recommendations for routine blood pressure measurement in children and adolescents. Findings can inform the development of EHR-based clinical decision support tools to augment blood pressure screening and recognition of prehypertension and hypertension in pediatric patients.

## Introduction

Obesity-related health conditions, including hypertension, are receiving increased attention (1). Less than 3% of US children have hypertension, although the prevalence of hypertension in obese children may be as high as 20% (1–4). Although the cardiovascular sequelae of hypertension are rarely experienced in childhood, evidence suggests that elevated blood pressure (BP) in childhood may predict increased cardiovascular risk in young adulthood (5,6).

The Task Force on the Diagnosis, Evaluation and Treatment of High Blood Pressure in Children and Adolescents (7–9) recommends that BP be measured in children 3 years or older at all health care visits. In addition, guidelines from both the American Academy of Pediatrics’ Bright Futures (10) and the Expert Panel of Integrated Guidelines for Cardiovascular Health and Risk Reduction in Children and Adolescents recommend annual BP screening in all children aged 3 to 17 years (11). A report that used data from the National Hospital Ambulatory Medical Care Survey found that BP was measured at only two-thirds of well-child visits and one-third of nonwell-child visits (12). This implies an ongoing level of noncompliance with recommendations for BP measurement among children. However, our understanding of factors that influence measurement of BP in children and adolescents is incomplete. Our objective was to identify factors associated with measurement of BP in primary care visits in 2 large medical groups with diverse patient populations.

## Methods

This study was conducted in 2 integrated health care delivery systems that together provide care for more than 1 million people: HealthPartners Medical Group in Minneapolis, Minnesota, and Kaiser Permanente Colorado in Denver, Colorado. EpiCare electronic health records (EHRs) were used at both sites, and data from the 2 health plans were restructured into a common, standardized format with identical variable names, definitions, labels, and coding. Study subjects were 105,634 patients aged 3 to 17 with 1 or more outpatient primary care visits (family practice or pediatrics) from January 1, 2007, through December 31, 2010, at either site. We excluded children with diagnosed hypertension (n = 1,382) and children taking anti-hypertensive medications (n = 559), because BP measurement recommendations differ for these patients. Thus, the final analytic cohort was reduced to 103,693 eligible subjects. The HealthPartners institutional review board reviewed, approved, and monitored the study.

The heights, weights, and BPs used in this study were measured during primary care visits as part of routine clinical care. BP measurement data from all visits to primary care outpatient clinics within the HealthPartners system or the Kaiser system were included for analysis. BP data from inpatient, urgent care, and specialty departments were excluded. Well-child visits were identified by the American Medical Association’s Current Procedural Terminology codes. All remaining primary care visits were defined as “nonwell-child.” Patient age and sex were obtained from membership databases. Race/ethnicity data were obtained from outpatient registration data or hospital discharge records and were available for 76% of subjects. BP was measured by trained staff predominantly using aneroid sphygmomanometers recalibrated as needed by bioengineering services. All measurements were conducted in the seated position with selection of cuff size appropriate to arm size. BP values, height, weight, and body mass index (BMI) percentiles were extracted from EHRs. Age- and sex-specific BMI percentiles were calculated by using Centers for Disease Control and Prevention equations (13). The number of clinic visits in last 12 months was compiled from EHR data.

Means, standard deviations, and frequencies were computed for patient- and visit-level characteristics. To determine the frequency of BP measurements at visits other than well-child visits, we conducted a multivariable analysis restricted to nonwell-child visits. We used mixed-model logistic regression with maximum likelihood estimation to compute predicted probabilities of BP measurement. Probability of BP measurement at nonwell-child visits was modeled as a dichotomous outcome adjusted for sex, age, BMI percentile, race/ethnicity, use (clinic visits per year), clinic department (pediatrics or family practice), and study site. Pairwise differences between categories were tested, and multiple comparisons were controlled using the Tukey-Kramer method in all multivariable models. We used a similar statistical model to examine the rate of annual BP measurement at any primary care visit by age group adjusted for study site. Analyses were performed with SAS Version 9.2 (SAS Institute, Inc).

## Results

Non–well-child visits comprised 70% of primary care visits (Table 1). BP was measured at 95% of well-child visits and 69% of nonwell-child primary care visits (*P* < .001).

**Table 1:** Characteristics of Female (N = 51,437) and Male (N = 52,256) Study Subjects and Health Care Visits, Minnesota and Colorado, 2007­–2010

Characteristic	Female^a^	Male^a^
**Age group, y**		
3–5	13,421 (26)	14,161 (27)
6–8	8,597 (17)	9,030 (17)
9–11	9,374 (18)	9,542 (18)
12–14	9,837 (19)	9,836 (19)
15–17	10,208 (20)	9,687 (19)
**Race/ethnicity**		
Non-Hispanic white	24,721 (48)	25,194 (48)
African American	6,861 (13)	6,833 (13)
Asian	2,812 (5)	2,780 (5)
Hispanic	4,229 (8)	4,218 (8)
Other/unknown^b^	12,814 (25)	13,231 (25)
**Body mass index (BMI) percentile**		
<85th	24,867 (48)	25,497 (49)
85th to <95th	5,062 (10)	55,095 (10)
≥95th	3,883 (8)	4,985 (10)
Missing BMI	17,625 (34)	16,679 (32)
**Government insurance**	4,577 (9)	4,503 (9)
**Visits**		
**Total visits, n**	256,122	246,496
**Visits by department type**		
Family practice	36,833 (15)	33,890 (14)
Pediatrics	213,112 (85)	206,528 (86)
**Visits by visit type**		
Well child	73,349 (29)	75,497 (31)
Nonwell child	176,596 (71)	164,921 (69)
**Visits with blood pressure measurement**		
Well child	69,716 (95)	71,650 (95)
Nonwell child	123,962 (70)	112,529 (68)

a Values are N (%) unless otherwise indicated. Percentages may not add to 100 because of rounding.

b Other race/ethnicity includes Native American (1% of total) and mixed (<1% of total).

Many factors influenced the probability of BP measurement at nonwell-child visits (Table 2). After adjusting for sex, age, BMI percentile, race/ethnicity, use, clinic department, calendar year, and study site, the percentage of nonwell-child visits that included BP measurement increased linearly with patient age (*P* < .001). Compared with non-Hispanic white children, other racial/ethnic groups had small but significantly higher percentages of visits with BP measurement. The probability of BP measurement was higher at family practice clinics (87%) than at pediatric clinics (73%, *P* < .001). Children with government-subsidized insurance were slightly more likely to have BP measured than those with commercial insurance (83% vs. 75%, *P* < .001). The proportion of children with an annual BP measurement during the observation period increased with age (Table 3). Overall, over 89% of children with any primary care visits had annual BP measurements. When BP was entered into the EHR in millimeters of mercury (mm Hg), 86% of the time it was converted to a BP percentile, primarily because a height measurement was not available to calculate the percentile.

**Table 2:** Percentage of Nonwell-Child Primary Care Visits With Documented Blood Pressure Measurements, Minnesota and Colorado, 2007–2010

Variable	Unadjusted %	*P* Value	Adjusted %^a^	*P* Value
**Sex**				
Female	73	[Reference]	76	[Reference]
Male	73	.002	76	.05
**Type of practice**				
Family	87	[Reference]	87	[Reference]
Pediatrics	70	<.001	73	<.001
**Age-group, y**				
3–5	44	[Reference]	49	[Reference]
6–8	66	<.001	69	<.001
9–11	74	<.001	76	<.001
12–14	81	<.001	82	<.001
15–17	88	<.001	90	<.001
**Race/ethnicity**				
Non-Hispanic white	73	[Reference]	74	[Reference]
African American	74	.34	81	<.001
Asian	68	<.001	77	.001
Hispanic	74	.97	76	.18
Other/unknown^b^	73	.98	77	<.001
**Body mass index percentile**				
<85th	80	[Reference]	85	[Reference]
85th to <95th	81	.55	84	.58
≥95th	82	.02	85	.99
Missing	63	<.001	65	<.001
**Visits per year**				
<1	74	[Reference]	76	[Reference]
1	73	<.001	76	.68
2 or 3	71	<.001	76	.82
≥4	72	<.001	77	.27
**Insurance type**				
Commercial	73	[Reference]	75	[Reference]
Government	75	<.001	83	<.001

a Adjusted for multiple comparisons. Includes sex, age group, body mass index category, race/ethnicity, department in which visit took place, use (visits per year), calendar year, and health system (HealthPartners or Kaiser Permanente Colorado).

b Other race/ethnicity includes Native American (1% of total) and mixed (<1% of total).

**Table 3:** Percentage of Children Receiving Annual Blood Pressure Measurements at Primary Care Visits, by Age, Minnesota and Colorado, 2007–2010^a^

Year	2007 (N = 61,133)	2008 (N = **62,526)**	2009 (N = **61,432)**	2010 (N = **53,367)**
**Age, y**				
3–5	81	80	77	76
6–8	87	86	88	88
9–11	91	91	91	90
12–14	94	94	94	94
15–17	94	94	94	95
**All age groups**	89	89	88	90

a Adjusted for study site.

## Discussion

Data from this study document high compliance with recommendations from the Task Force on the Diagnosis, Evaluation and Treatment of High Blood Pressure in Children and Adolescents (7–9) that BP measurement should be part of the clinical examination, particularly at well-child visits. However, only 69% of nonwell-child primary care visits included BP measurements. This may be due, in part, to multiple nonwell-child visits in a single episode of care (eg, return visits for otitis media) or to nonwell-child visits occurring in close succession to previous well-child visits. Although BP measurement at nonwell-child visits was 69%, in this cohort of children and adolescents with primary care visits, the proportion of subjects who had at least 1 BP measurement annually was high.

Other studies have reported BP measurement at only 35% of pediatric visits and 67% of well-child visits (12). The rates observed in our study are considerably higher, perhaps because the data in this study are more recent and may reflect increased adoption of clinical guidelines in recent years or because data were derived from 2 large health systems that have standardized rooming procedures.

Several factors influence the likelihood of BP measurement at nonwell-child primary care visits. BP measurement frequency was lowest in the preschool age group, possibly due to an expectation that few younger children have hypertension. Surprisingly, despite the well-known association of overweight and obesity with hypertension, children with a BMI at or above the 85th percentile did not have BP measured more often than children with a BMI below the 85th percentile, perhaps because providers may not recognize overweight as often as obesity in children and adolescents (14,15). More likely, at primary care visits for minor illness, providers may not be thinking about hypertension. Finally, family practice clinics measured BP more often than pediatric clinics, possibly because pediatricians are more likely to adhere to the Bright Futures guidelines, which recommend annual BP screenings, or because family physicians and their rooming nurses are accustomed to measuring BP in most patients (10). This finding may be explained by our observation that family physicians tend to see more adolescents and fewer young children than pediatricians.

Although data from various studies suggest deficits in the quality of care provided to children (16) and adults (17), our EHR-derived data indicate that providers measure BP on nearly all visits with children and adolescents seen in the clinic at least once a year, as recommended by the Bright Futures guidelines. However, interpretation of BP measures in pediatric populations is complicated by age-, sex-, and height-specific BP criteria for hypertension and requires multiple measurements. Although our data indicate that the rate of BP measurement is high, it is unknown if BP data are being properly interpreted to correctly assign a diagnosis of prehypertension or hypertension when diagnostic criteria are met.

Many factors limit the interpretation of our data. First, to be included in the study population, a child had to have at least 1 primary care visit during the observation period; as a result, the BP measurement rates appear to be high because children without visits, and therefore without BP measurements, are not included. Second, it was beyond the scope of these analyses to examine charts and progress notes for additional BP measurements or documented reasons for nonmeasurement. Third, the study population may not be representative. Although the study population includes predominantly insured patients, it also includes many patients receiving government-subsidized health insurance, with substantial diversity in race/ethnicity and socioeconomic status (18). These data from integrated health systems may not reflect BP measurement practices in other care settings.

The results from this large cross-sectional study show good adherence to national recommendations for routine annual BP measurement in children (8,10,11). Although BP was measured at only 69% of nonwell-child visits, among those with primary care visits, 89% had a minimum of 1 BP measurement per year regardless of visit type (well-child or nonwell-child) and visit frequency, which is in accordance with the Bright Futures guidelines (10). Whether the high rate of measurement translates to timely and complete recognition of hypertension requires additional study. These findings can inform the development of EHR-based clinical decision support tools to augment BP screening and recognition of prehypertension and hypertension in pediatric patients by presenting BP in percentiles, rather than in mm Hg, as well as display recent BPs.
